# Dependency of the regio- and stereoselectivity of intramolecular, ring-closing glycosylations upon the ring size

**DOI:** 10.3762/bjoc.7.189

**Published:** 2011-12-01

**Authors:** Patrick Claude, Christian Lehmann, Thomas Ziegler

**Affiliations:** 1 Institute of Organic Chemistry, University of Tübingen, Auf der Morgenstelle 18, 72076 Tübingen, Germany; 2 Institute for Organic Chemistry, University of Lausanne BCH-Dorigny, CH-1015 Lausanne, Switzerland

**Keywords:** intramolecular glycosylation, molecular modeling, prearranged glycosides

## Abstract

Phenyl 3,4,6-tri-*O*-benzyl-2-*O*-(3-carboxypropionyl)-1-thio-β-D-galactopyranoside (**1**) was condensed via its pentafluorophenyl ester **2** with 5-aminopentyl (**4a**), 4-aminobutyl (**4b**), 3-aminopropyl (**4c**) and 2-aminoethyl 4,6-*O*-benzylidene-β-D-glucopyranoside (**4d**), prepared from the corresponding *N*-Cbz protected glucosides **3a**–**d**, to give the corresponding 2-[3-(alkylcarbamoyl)propionyl] tethered saccharides **5a**–**d**. Intramolecular, ring closing glycosylation of the saccharides with NIS and TMSOTf afforded the tethered β(1→3) linked disaccharides **6a**–**c**, the α(1→3) linked disaccharides **7a**–**d** and the α(1→2) linked disaccharide **8d** in ratios depending upon the ring size formed during glycosylation. No β(1→2) linked disaccharides were formed. Molecular modeling of saccharides **6**–**8** revealed that a strong aromatic stacking interaction between the aromatic parts of the benzyl and benzylidene protecting groups in the galactosyl and glucosyl moieties was mainly responsible for the observed regioselectivity and anomeric selectivity of the ring-closing glycosylation step.

## Introduction

**Figure 1:**
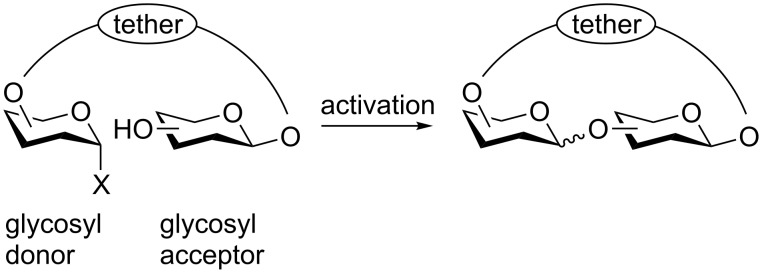
Schematic representation of the “prearranged-glycoside concept” for intramolecular, ring-closing glycosylation.

Intramolecular *O*-glycosidic bond formation of tethered glycosyl donors and acceptors (prearranged glycosides) resembles to some extent enzyme-catalyzed glycosylation reactions where the glycosyl donor and glycosyl acceptor are first bound in the active site of an enzyme and thus, the glycosidic bond forms intramolecularly. Three different concepts for the intramolecularization of glycosylation reactions have been studied so far. For recent reviews on this subject see [1–4]. In the “leaving-group-based concept”, the glycosyl acceptor is attached to the leaving group of the glycosyl donor and *O*-glycosidic bond formation occurs synchronously to the cleavage of the leaving group [5–9]. In the “aglycon-delivery concept”, the glycosyl acceptor is attached to a labile acetal [10–14] or silylene group [15–17], which is cleaved and the glycosyl acceptor is “delivered” to the anomeric center upon its activation. In the “prearranged-glycoside concept”, the sole true intramolecular glycosylation approach which was developed in our [18–20] and Valverde’s group [21], glycosyl donor and acceptor are linked by a stable tether attached to positions not directly involved in the glycosylation step. Upon activation of the leaving group, an intramolecular, ring-closing condensation occurs affording a tethered saccharide (Figure 1).

Despite the fact that the “prearranged-glycoside concept” for intramolecular glycosylation has been successfully applied to the construction of glycosidic bonds that are otherwise difficult to establish (i.e., β-D-mannosidic and β-L-rhamnosidic bonds) and to the synthesis of complex oligosaccharides [22–24], the distinct effects which govern the regio- and stereoselectivity of the intramolecular formation of glycosidic bonds remain enigmatic. Both, the nature and the torsional flexibility of the tether and the tethered positions of the glycosyl donor and acceptor, as well as the size of the ring (that forms during the intramolecular glycosylation step), the solvent, and the activation procedure all seem to play a crucial role in this respect [3,25–26]. Previously, we have also shown for intramolecular mannosylations that double diastereodifferentiation is responsible in part for the anomeric selectivity of such intramolecular glycosylations, although the exact cause of this effect has not been unambiguously identified so far [27]. Therefore, we prepared a series of prearranged glycosides constructed out of a 1-thio-galactosyl donor and a 4,6-*O*-benzylidene-glucose acceptor tethered by peptide-bond-containing linkers of variable size, in order to study systematically the parameters influencing the outcome of the intramolecular, ring-closing glycosylation step. In order to further provide a rationalization for the observed regio- and stereochemistry of the reaction, we also performed a molecular modeling study, applying a force-field that was developed for general use in organic and pharmaceutical chemistry [28–30].

## Results and Discussion

**Figure 2:**
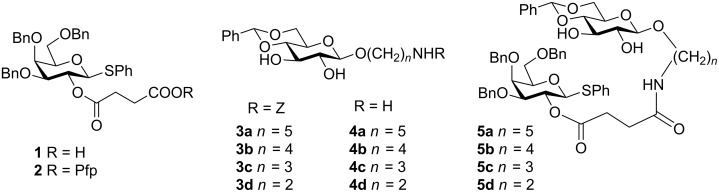
Structures of compounds **1**–**5**.

For the preparation of the prearranged glycosides **5**, we started from phenyl 3,4,6-tri-*O*-benzyl-2-*O*-(3-carboxypropionyl)-1-thio-β-D-galactopyranoside (**1**), which was prepared in three steps from 3,4,6-tri-*O*-benzyl-1,2-*O*-methoxyethylidene-α-D-galactopyranoside as previously described [31] and condensed with pentafluorophenol (dicyclohexylcarbodiimide, ethyl acetate, 0 °C) to afford the pentafluorophenyl ester **2** in 87% yield. Aminoalkyl 4,6-*O*-benzylidene-β-D-glucopyranosides **4** were prepared from the corresponding Z-protected glucosides **3**. Previously, we prepared 5-(benzyloxycarbonylamino)pentyl 4,6-*O*-benzylidene-β-D-glucopyranoside (**3a**) by acetalation of 5-(benzyloxycarbonylamino)pentyl β-D-glucopyranoside with benzaldehyde and ZnCl_2_ [32]. Here, we used the more convenient method for the acetalation step with benzaldehyde dimethyl acetal (PhCH(OMe)_2_, cat. TsOH, MeCN, 25 °C) [33], which gave **3a** in 71% yield. Selective removal of the Z group from **3a** was a rather delicate task because the benzylidene group must remain unaffected. After careful optimization, hydrogenation of **3a** with Lindlar catalyst (Pd on BaCO_3_) in ethanol at room temperature gave **4a** in 91% yield, which was sufficiently pure to be used in the next step without further purification. No hydrogenolysis of the benzylidene group was observed under these conditions. Compound **3b** has not been described in the literature so far. It was prepared by first glucosylating 4-(benzyloxycarbonylamino)butanol [34] with 2,3,4,6-tetra-*O*-benzoyl-α-D-glucopyranosyl trichloroacetimidate [35] (cat. TMSOTf, CH_2_Cl_2_, −10 °C, 2 h, 68%). Next, Zemplén deacylation of the intermediate glucoside (cat. NaOMe, MeOH, 25 °C, 16 h, 97%) and acetalation as described for compound **3a** afforded compound **3b** in 77% yield. Hydrogenation of the latter with Lindlar catalyst then gave **4b** in 80% yield. Similarly, compounds **4c** and **4d** were prepared by hydrogenolysis of the known Z-protected glucosides **3c** [33] and **3d** [36]. Finally, compounds **4a**–**d** were condensed with pentafluorophenyl ester **2** (ethyl acetate, 25 °C, 16 h) to afford the prearranged glycosides **5a**–**d** in 78–89% yield (Figure 2). As the condensation of ester **2** and amines **4** progressed, the products **5** precipitated from the solvent due to their poor solubility in ethyl acetate. This, however, facilitated the workup of the reaction mixtures significantly, as the prearranged glycosides **5** was isolated by simple filtration and purified by recrystallization.

**Scheme 1:**
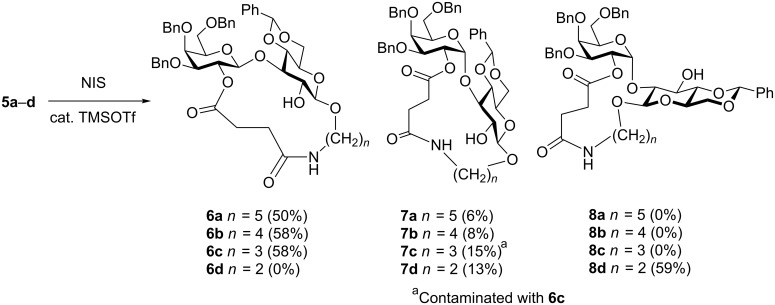
Intramolecular, ring-closing glycosylation of prearranged glycosides **5a**–**d**.

All prearranged glycosides **5** were intramolecularly glycosylated by activating the phenylthio group with the NIS-TMSOTf reagent (Scheme 1) [37]. Attention had to be paid to the choice of solvent, because the tethered glycosides **5** were only poorly soluble in most solvents that are usually applied for glycosylations with thioglycosides under activation with NIS. Best results were obtained with a 1:1 mixture of CH_2_Cl_2_ and MeCN. All intramolecular couplings proceeded smoothly at −5 °C within one hour. The products **6**−**8** were purified by conventional chromatography on silica gel with mixtures of CH_2_Cl_2_ and acetone as the eluent.

**Scheme 2:**
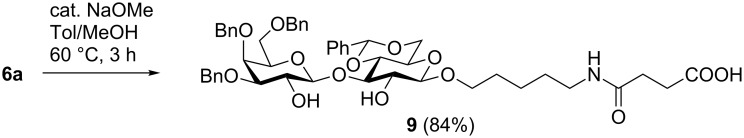
Ring opening of compound **6a**.

For the prearranged glycoside **5a**, only the β(1→3)-linked product **6a** (50%) and the α(1→3)-linked product **7a** (6%) were obtained upon intramolecular glycosylation, forming an 18-membered macrocyclic ring. No (1→2)-linked disaccharides **8** were detected. The structures of **6a** and **7a** were unambiguously assigned by NMR spectroscopy. In compound **6a** C-1 of the galactosyl residue resonated at 103.2 ppm, indicating a β-linkage, while in compound **7a** it resonated at 96.6 ppm, indicating an α-linkage. The (1→3)-linkage for both disaccharides was proven by HMBC NMR experiments, which revealed a weak ^3^*J*-CH-correlation between H-1 of the galactosyl residues and C-3 of the glucosyl residues. The structure of **6a** was further proven by the opening of the peptide bridge at the ester function, to give β(1→3)-linked disaccharide **9** in 84% yield (Scheme 2). Similar results were obtained for the intramolecular glycosylation of prearranged glycoside **5b**, which afforded the β(1→3)-linked 17-membered macrocyclic ring **6b** in 58% yield and the corresponding α(1→3)-linked ring **7b** in 8% yield. Again, no (1→2)-linked disaccharides **8** were found in this case, and the structures of **6b** and **7b** were unambiguously assigned by NMR spectroscopy showing C-1 of the galactose residue at 102.8 ppm for **6b** and at 94.7 ppm for **7b**. HMBC NMR experiments revealed ^3^*J*-CH-correlation between C-1 of the galactosyl residues and H-3 of the glucosyl residues. For the prearranged glycoside **5c**, the results for the intramolecular 16-membered ring-forming glycosylation were also similar. Only the β(1→3)-linked disaccharide **6c** (58%) and the α(1→3)-linked disaccharide **7c** (15%) were obtained, although **7c** was contaminated with traces of **6c**. However, the structures of both **6c** and **7c** were again unambiguously assigned by NMR spectroscopy showing C-1 of the galactose residue at 102.7 ppm for **6c** and at 95.4 ppm for **7c**, and significant weak ^3^*J*-CH-correlations between H-1 of the galactosyl residue and C-3 of the glucosyl residue in **6c** and C-1 of the galactosyl residue and H-3 of the glucosyl residue in **7c**.

**Figure 3:**
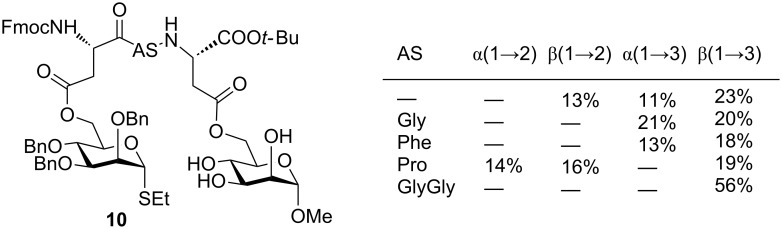
Intramolecular glycosylation of peptide-tethered mannosides according to Fairbanks [38–40].

Surprisingly, the prearranged glycoside **5d** gave no β(1→3)-linked disaccharide **6d** and only a small amount (13%) of the corresponding α(1→3)-linked 15-membered ring **7d**. Instead, the 14-membered α(1→2)-linked ring **8d** was formed as the major product and was isolated in 59% yield. Here, no β(1→2)-linked product was detected. The α(1→2)-linkage for **8d** was evident from its NMR spectra showing a signal at 97.3 ppm for C-1 of the galactosyl residue and a weak ^3^*J*-CH-correlation between C-1 of the galactosyl residue and H-2 of the glucosyl residue. Likewise, compound **7d** showed C-1 of its galactosyl residue resonating at 97.2 ppm and a weak ^3^*J*-CH-correlation between C-1 of the galactosyl and H-3 of the glucosyl residue. The results obtained in this study mirror to some extent the trend observed by Fairbanks for the intramolecular mannosylation of compound **10**, where the donor and the acceptor were tethered at their 6-positions by di- through to tetrapeptides (Figure 3) [38–40]. As observed in our cases, the size of the ring that forms during intramolecular glycosylation influences the anomeric outcome of the glycosylation. However, the distinct regio- and stereoselectivities in such cases are also strongly influenced by the nature of the tether and thus, by the preferred conformations that the prearranged glycosides adopt during the ring-closing condensation. Likewise, Warriner showed that the anomeric selectivity of an intramolecular glycosylation between a glycosyl donor and an acceptor that are tethered by a specially designed tripeptide through their respective 6-OH groups, depends on the peptide tether (peptide-templated saccharide synthesis) [41–42]. Therefore, in order to understand these factors better, we performed a molecular modeling study for the intramolecular glycosylation of the prearranged glycosides **5** and their cyclization products **6**–**8**.

### Molecular modeling studies

**Figure 4:**
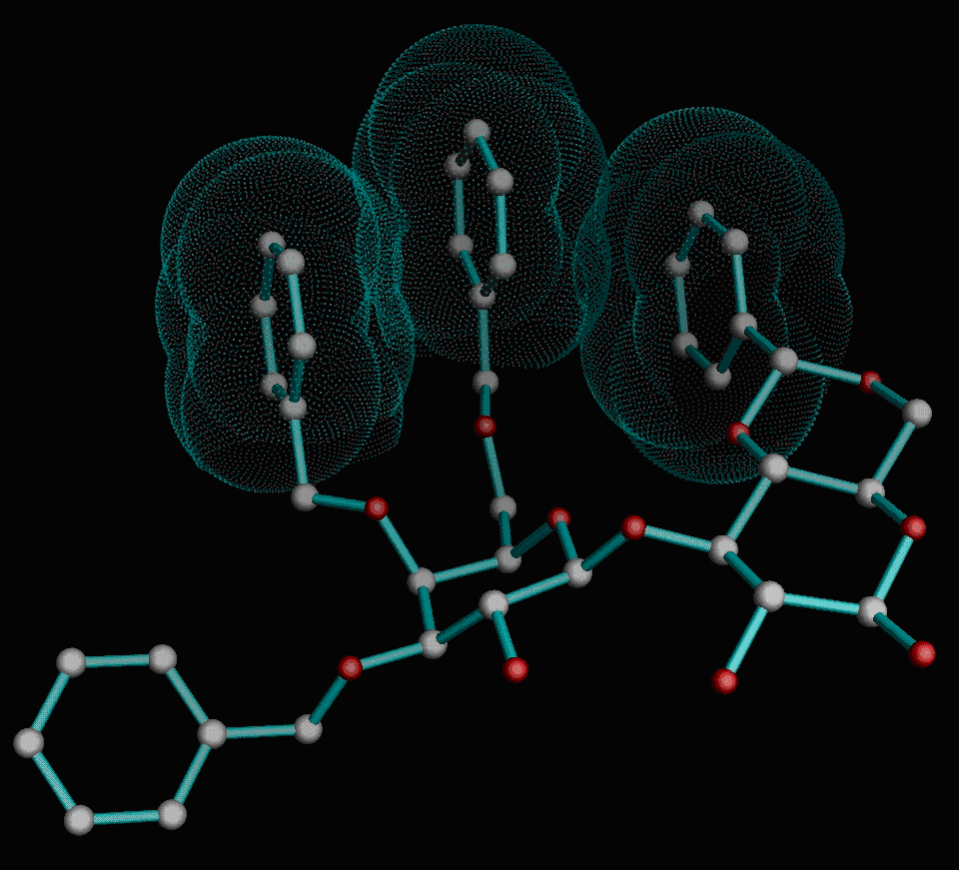
Isolated β(1→3)-glycosidic linkage favored by triad aromatic-stacking interactions (ASI).

Molecular models of the intramolecular glycosylation products **6–8** were built by using the molecular modeling program suite Moloc [28–29]. First, the β(1→3)-selective cyclization junction was modelled and energy minimized in a recently reparametrized version of the MAB force field [30], revealing that a strong aromatic triad is formed between the 4,6-*O*-benzylidene group of the glucose moieties and the 4- and 6-*O*-benzylether groups of the adjacent galactose residues (Figure 4).

**Figure 5:**
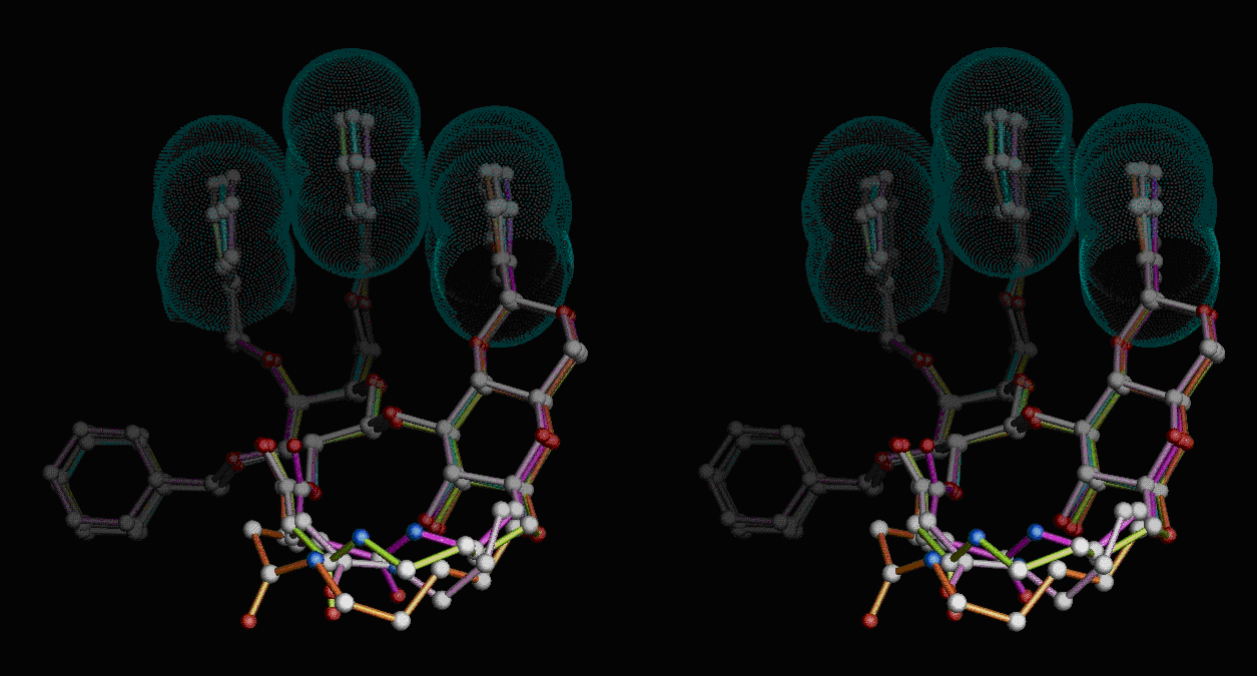
Stereo view of the superposition of β(1→3)-linked disaccharide models of **6a–d** in stable triad-ASI conformations. **6a** (*n* = 5) orange, **6b** (*n* = 4) green, **6c** (*n* = 3) pink, **6d** (*n* = 2) magenta.

Further, the main glycosylation products **6–8** for interlinking chains (CH_2_)*_n_* with *n* = 5, 4, 3, 2 were constructed and energy minimized (Figure 5). A strain-free β(1→3)-junction is possible for all constitutions, whereby the family of conformations (including the case *n* = 2) is compatible with the aforementioned stacking effect. It is expected, that the hydroxy group at position 2 of disaccharide **6a** is buried within the ring, and thus, is less reactive and hence difficult to acylate or to glycosylate: Indeed, all attempts to benzoylate the disaccharide **6a** under various conditions failed.

**Figure 6:**
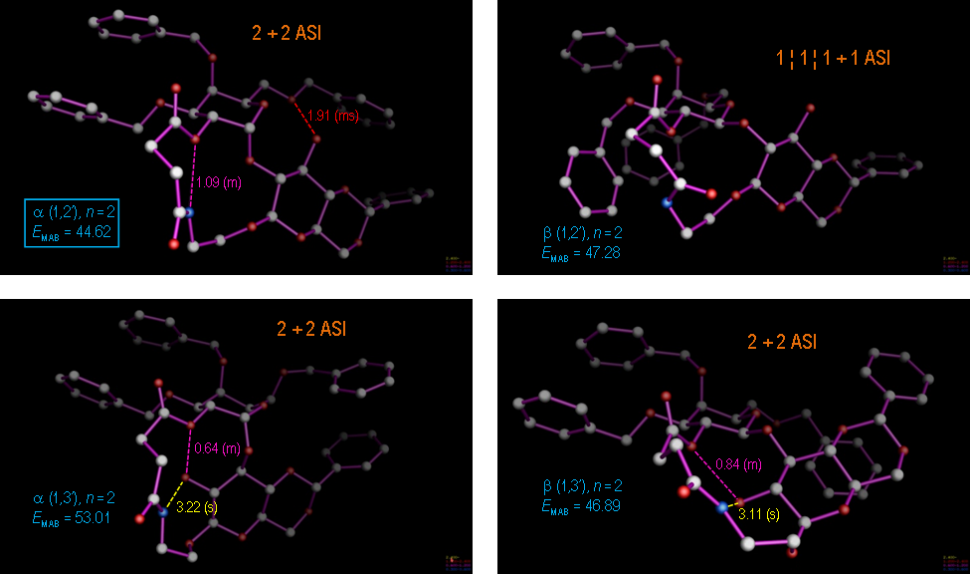
Most-stable product conformations for the cyclo-glycosidation reaction with ring size spacer *n* = 2 (constitutions **6d**, **7d**, **8d**, and further the isomer β(1→2), were not observed). The various regio- and stereoisomers were modeled and subjected to molecular dynamics (MD) runs of at least 5000 ps, wherefrom the observed major diastereomer **8d** emerged with a lowest free energy by more than 2.2 kcal/mol with respect to the β(1→3) isomer **6d**.

In spite of this favourable arrangement, for the case *n* = 2 predominant formation of the α(1→2)-linked product **8d** was observed, justifying a more profound conformational analysis of this product (Figure 6). Conformational molecular dynamics runs (MD) were performed on both anomers of the two regioisomers of **8d** to simulate the behavior of the molecule during more than 5000 ps of molecular motion. Thereby, it was found that for the observed regio- and stereochemistry an alternative conformation with two ASIs (2 + 2 ASI) and two intramolecular hydrogen bonds is significantly (2.2 kcal/mol) more stable than the virtual β(1→3)-linked product **6d**; however, the triad-stabilized starting conformation for the MD is energetically significantly higher (4.8 kcal/mol) than the MD optimized conformation, since the latter profits from two hydrogen bonds and also two ASIs, even though it is decoupled. Thus, for the product constitution **8d** with the highest relative macrocyclic ring constraint, the observed regio- and stereoselectivity can be related with intra- and interresidual molecular hydrogen bonding and non-bonding ASI terms in the most stable product conformation.

Mechanistically, the complex reaction is thought to be initiated by a fast [43], exergonic glycosyl cation formation, which is then followed by the comparatively slow, entropy-reducing intramolecular cyclization step. Within the frame of an overall S_N_1 reaction, the cyclization step is regarded as the rate-determining step; therefore, the transition state for the nucleophilic addition to the glycosyl cyclic oxonium intermediate may allow for a rationalization of the observed regioselectivity over the course of the reaction. If the rate-determining step of the reaction is endergonic, then differences in productlike transition states will account for the observed selectivity according to Hammond’s postulate [44].

**Figure 7:**
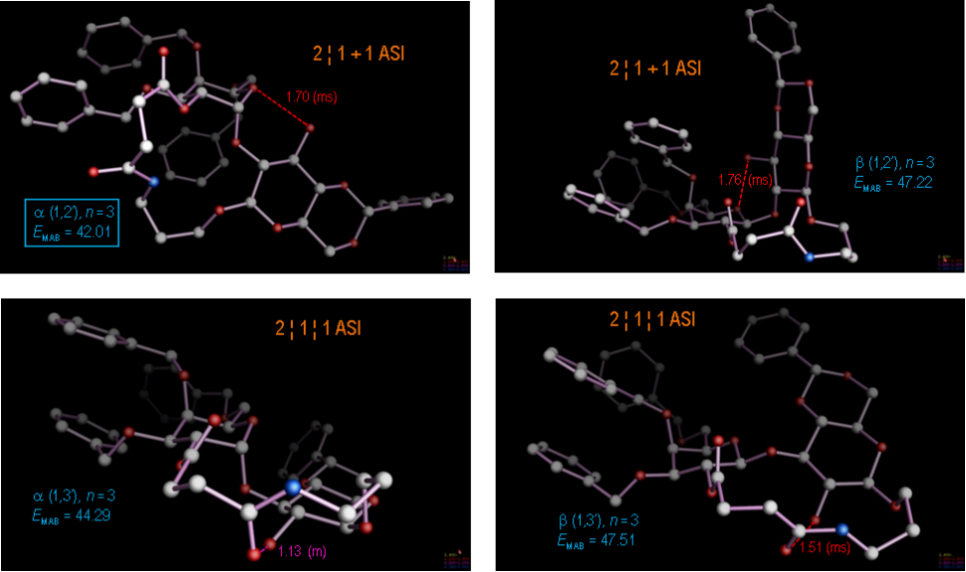
Most-stable product conformations for the cyclo-glycosidation reaction with ring size spacer *n* = 3 (constitutions **6c**, **7c**, **8c**, and further the isomer β(1→2), were not observed). As in Figure 6, the various regio- and stereoisomers were modeled and subjected to molecular dynamics (MD) runs of at least 5000 ps, wherefrom the observed major diastereomer **8c**, α(1→2), in its lowest conformation was again obtained with a free energy that was 2.3 kcal/mol lower than that for the next highest diastereomer **7c**, α(1→3).

**Figure 8:**
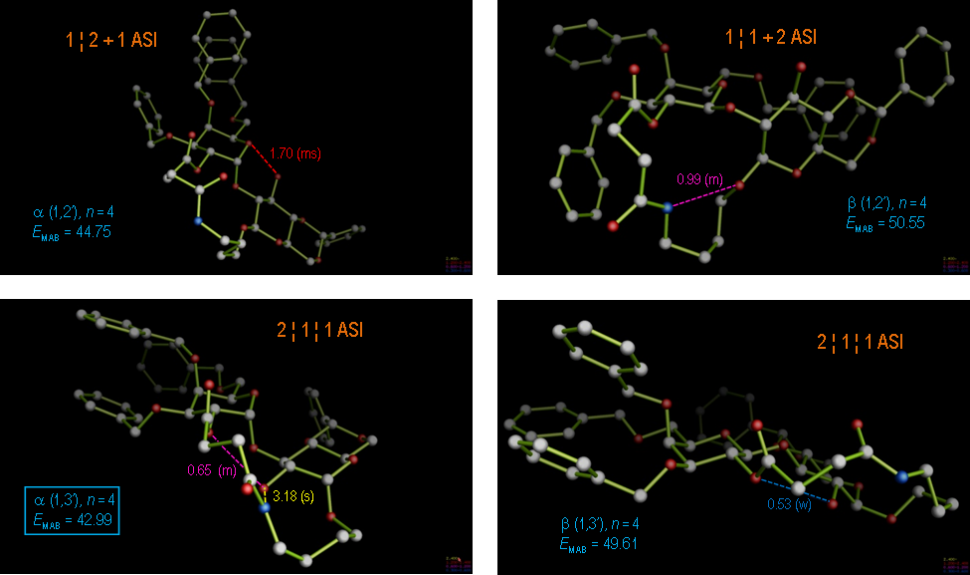
Most-stable product conformations for the cyclo-glycosidation reaction with ring size spacer *n* = 4 (constitutions **6b**, **7b**, **8b**, and further the isomer β(1→2), were not observed). As in Figure 6, the various regio- and stereoisomers were modeled and subjected to molecular dynamics (MD) runs of at least 5000 ps, wherefrom the observed major diastereomer **7b**, α(1→3), in its lowest conformation was obtained with a free energy that was 1.8 kcal/mol lower than that for the next highest diastereomer **8b**, α(1→2).

**Figure 9:**
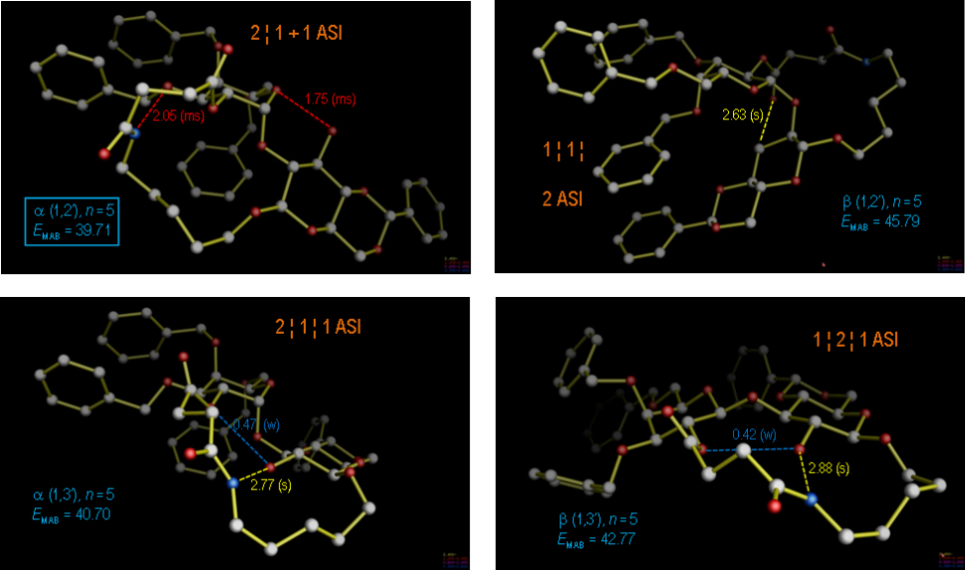
Most-stable product conformations for the cyclo-glycosidation reaction with ring size spacer *n* = 5 (constitutions **6a**, **7a**, **8a**, and further the isomer β(1→2), was not observed). As in Figure 6, the various regio- and stereoisomers were modeled and subjected to molecular dynamics (MD) runs of at least 5000 ps, wherefrom the observed major diastereomer **8a**, α(1→2), in its lowest conformation invariably emerged with a free energy that was 1.0 kcal/mol lower than that for the next higher diastereomer **7a**, α(1→3).

For the reaction path to the most constrained macrocylic ring system (constitution series **d** with *n* = 2), this argument holds, even to the extent of regarding the most stable product conformation as a valid model for the transition state: The observed major diastereomer **8d**, α(1→2), emerges as the most stable product conformation by more than 2.2 kcal/mol compared to its next highest diastereomer **6d**, β(1→3), as documented in Figure 6. Nevertheless, a closer approximation to the real transition state involving the pyranose cyclic oxonium intermediate is possible within our calibrated force field approach: Figure 7 highlights the stereoelectronic course of the reaction, which involves the 2’-OH nucleophilic center in a favorable, 1,2-diaxial addition to the cyclic C=O(+) moiety, resulting in the α(1→2)-anomer. This reaction path not only leads directly to the establishment of the most-stable, chair conformation in the pyranose ring, but moreover proves to be entirely consistent with accepted stereoelectronic principles in organic chemistry [45–47]. Given these arguments, the obvious question for the alternative stereochemical outcome of the cyclo-glycosidation reaction for the cases *n* = 3, 4 and 5 (series **c**, **b** and **a**) arises: In terms of the canonical stereochemical principles, we do not perceive a clear reason why they should not also cyclize to the α(1→2)-stereoisomer; indeed, extensive MD calculations (>5000 ps) on each of the possible product diastereomers (Figure 7, Figure 8 and Figure 9) converge into most-stable, rearranged product conformations with most having the α(1→2)-stereochemistry.

**Figure 10:**
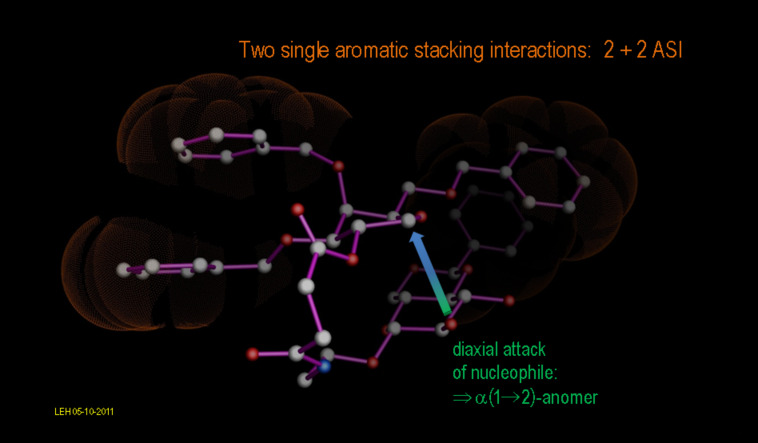
Canonical stereoelectronic course of the intramolecular glycosylation reaction leading to the α(1→2)-linked product **8d** favored by two independent aromatic-stacking interactions (2 + 2 ASI). Notably, the diaxial attack of the nucleophile in this case allows the direct formation of a chair conformation out of the pyranose cyclic oxonium intermediate.

As summarized in Table 1, the most stable product conformations are generally supportive of the formation of the α(1→2) stereo- and regiochemistry, which would occur over a transition state as depicted in Figure 10 for the case *n* = 2. For the comparatively unconstrained cases *n* > 2 our analysis appears thus to have failed, since for these cases the β(1→3) isomers have predominantly been isolated from the reaction. Can we nevertheless find a stringent, modeling-based argument for the alternative course of the reaction with extended linker geometries?

**Table 1:** Calculated free energies of the most stable isomers of compounds **6**–**8** and the isomers not found during cyclization of compounds **5**.

Compounds	Length of (CH_2_)*_n_* group^a^	Energy [kcal∙mol^−1^] of the most stable isomers^b^			
		**6a**–**d** β(1→3)	**7a**–**d** α(1→3)	**8a**–**d** β(1→2)^c^	**8a**–**d** α(1→2)

**6**–**8a**	*n* = 5	42.77	40.76	45.79^c^	39.70^c^
**6**–**8b**	*n* = 4	49.61	42.99	50.55^c^	44.75^c^
**6**–**8c**	*n* = 3	47.51	44.29	47.22^c^	42.01^c^
**6**–**8d**	*n* = 2	46.89^c^	53.01	47.28^c^	44.62

^a^See Scheme 1; ^b^Energies were calculated by molecular dynamics, >5000 ps, for each isomer. Individual aromatic stacking patterns and H-bond contributions in the respective product conformations are illustrated in composite Figures 6–9; ^c^Isomers not obtained during the intramolecular cyclization of compounds **5a**–**d**.

**Figure 11:**
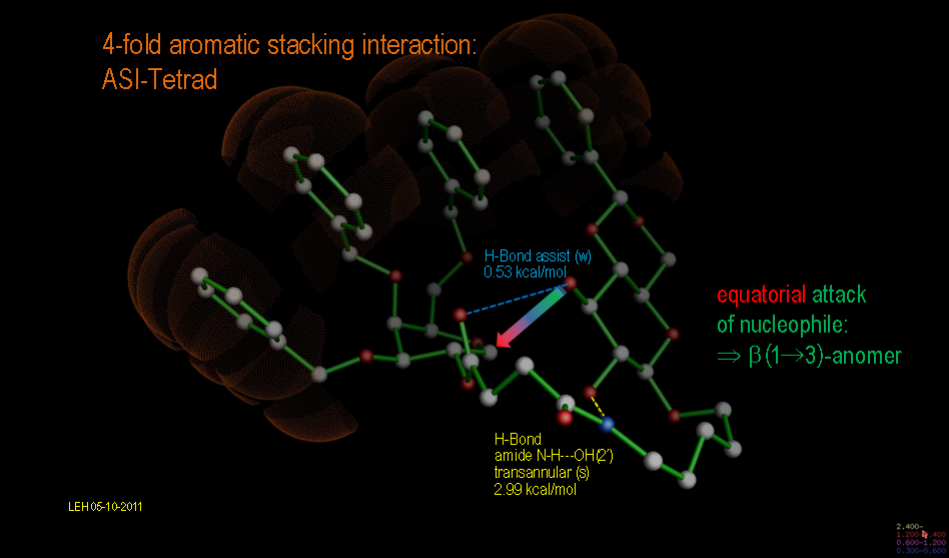
Unusual stereoelectronic course of the intramolecular glycosylation reaction leading to the β(1→3)-linked products **8a–c** induced by a cumulated tetrad aromatic-stacking interaction (4 ASI), and further by a strong transannular amide N–H···OH(2’) hydrogen bond (yellow) as well as an assisting neighboring H-bond (3’)O–H···O=C(ester) (blue). Extended MD runs (>10.000 ps) do not lead to a dissociation of the relayed aromatic tetrad.

Figure 11 illustrates what we intuitively anticipated at the beginning of the discussion, namely that the aromatic stacking interactions have a remarkable influence on the course of an intramolecular cyclization reaction: If the precyclized (seco) cyclic oxonium ion is modeled as an approximation for its transition state, the established stereoelectronic principles are overruled not only by a triple ASI, but rather by a *quadruply aligned stack: A tetrad ASI.* In extended MD runs (>10,000 ps), the tetrad does not dislocate into aggregates with alternate stacking patterns, whereas the corresponding constitution with a shorter tether bridge *n* = 2 rearranges after a short time of MD. The “non-canonical” tetrad ASI transition state is further stabilized by a strong transannular amide N–H···OH(2’) hydrogen bond as well as by an assisting, neighboring H-bond (3’)O–H···O=C(ester), which renders the previous more nucleophilic. In this case, the attack occurs from the opposite side of the oxonium intermediate and leads to stable product conformation only after formation of an initial pyranose envelope/boat conformation, which however easily flips to the chair conformation with the anomeric hydrogen in the axial and the glycosidic oxygen in the equatorial orientation.

Similar, possibly less-spectacular cases of product-controlling ASI effects have notably been found in peptide cyclization reactions [48–49] and very generally for the case of the induction of handedness in nucleic acid constructs ([50] and references cited therein). A remarkable differentiation of the regioselectivity of acylation reactions at differently exposed hydroxy groups was found for chiral 2’-*O*-tetrahydropyranyl nucleotides [51], whereby a particular hydroxy group is significantly deshielded in ^1^H NMR and consequently its nucleophilicity is thought to be enhanced by intramolecular hydrogen bonding.

## Conclusion

We showed, for the intramolecular glycosylation reaction via “prearranged” glycosides, that the stereo- and regioselectivity of this condensation reaction does not only depend upon the relative configuration of the involved hydroxyls of the glycosyl donor and acceptor, as was previously anticipated. Rather, in addition to these factors, interactions (π-stacking) of the protecting groups in close proximity to, as well as distant from, the reaction centers play an important role in determining the stereoselectivity of intramolecular glycosylations.

## Supporting Information

File 1 Experimental data.
