# Supplementary material for: Dependency of the regio- and stereoselectivity of intramolecular, ring-closing glycosylations upon the ring size
Source: Beilstein J Org Chem. 2011 Dec 1;7:1609–19. doi: 10.3762/bjoc.7.189 (PMC3252864; doi:10.3762/bjoc.7.189)
Supplement: File 1 — Experimental data. [file Beilstein_J_Org_Chem-07-1609-s001.pdf]

Supporting Information
for

Dependency of the regio- and stereoselectivity of intramolecular, ring-closing glycosylations upon the ring size

Patrick Claude[1], Christian Lehmann[2] and Thomas Ziegler[*1]

Address: [1]Institute of Organic Chemistry, University of Tuebingen, Auf der Morgenstelle 18, 72076 Tuebingen, Germany and [2]Institute for Organic Chemistry, University of Lausanne BCH-Dorigny, CH-1015 Lausanne, Switzerland

Email: Thomas Ziegler[*] - thomas.ziegler@uni-tuebingen.de; Christian Lehmann - lehmannc.3d@a3.epfl.ch

* Corresponding author

Experimental Data

General

All solvents were dried and distilled prior to their use. Reactions were performed under Ar and monitored by TLC on Polygram Sil G/UV silica gel plates from Machery & Nagel. Detection was effected by charring with H_2SO_4 (5% in EtOH) or by inspection of the TLC plates under UV light. NMR spectra were recorded on a Bruker ARX 250 spectrometer at 100 MHz for proton spectra and at 62.9 MHz for carbon spectra, and on a Bruker Avance 400 spectrometer at 400 MHz for proton spectra and at 100 MHz for carbon spectra. Tetramethylsilane was used at the internal standard. ESI-HRMS was performed on a Bruker FT-ICR spectrometer. FAB-HRMS was performed on a Finnigan MAT 65 spectrometer. Elemental analyses were performed on a Hekatech Euro 3000 CHN analyzer. Optical rotations were measured with a Perkin-Elmer Polarimeter 341. Preparative chromatography was performed on silica gel (0.032–0.063 mm) from Machery & Nagel with different mixtures of solvents as eluent.

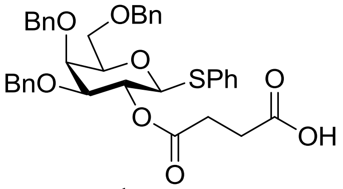

1

Phenyl 3,4,6-Tri-*O*-benzyl-2-*O*-(3-carboxypropionyl)-1-thio-β-D-galactopyranoside (1)

A solution of phenyl 3,4,6-tri-*O*-benzyl-1-thio-β-D-galactopyranoside [1] (11.5 g, 22 mmol), succinic anhydride (22 g, 0.22 mol) and a catalytic amount of DMAP (ca. 20 mg) in pyridine (270 mL) was stirred at 100 °C for 22.5 h. The mixture was concentrated and coevaporated with toluene. The residue was dissolved in CH_2Cl_2 (300 mL), washed successively with ice-cold 1 N aqueous HCl solution (3 × 100 mL) and saturated aqueous $NaHCO_3$ solution (2 × 100 ml), dried with Na_2SO_4, filtered and concentrated. Chromatography (toluene/acetone 8:1 v/v containing 0.1 vol % acetic acid) of the residue afforded **1** (8.3 g, 60%) as a colorless foam: $[\alpha]_D$ +12.5 (*c* 1.0, chloroform); ^{1}H NMR ($CDCl_3$) δ 7.49–7.00 (m, 20H, Ph-H), 5.39 (t, 1H, $J_{1,2}$ = 9.7 Hz, $J_{2,3}$ = 9.7 Hz, H-2), 4.86 (d, 1H, J = −11.6 Hz, CH_2Ph), 4.61–4.32 (m, 6H, H-1, CH_2Ph), 3.92–3.50 (m, 5H, H-3,4,5,6a,6b), 2.56–2.53 (m, 4H, CH_2); ^{13}C NMR ($CDCl_3$) δ 177.8 (COOH), 170.9 (COO), 86.6 (C-1), 81.4 (C-3), 77.6 (C-5), 74.4, 73.6, 72.1 (CH_2Ph), 73.0 (C-4), 70.0 (C-2), 68.9 (C-6), 29.1, 28.9 (CH_2); Anal. calcd for $C_{37}H_{38}O_8S$ (642.8): C, 69.14; H, 5.96; found: C, 68.99; H, 5.94.

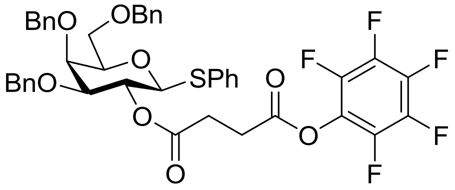

2

Phenyl 3,4,6-Tri-*O*-benzyl-2-*O*-(3-pentafluorophenoxycarbonylpropionyl)-1-thio-β-D-galactopyranoside (2)

Dicyclohexyl carbodiimide (2.74 g, 13.3 mmol) was added at 0 °C to a solution of compound **1** (8.3 g, 13.3 mmol) and pentafluorophenol (2.44 g, 13.4 mmol) in ethyl acetate (120 mL), and the mixture was stirred at 0 °C for 12 h. The mixture was warmed to room temperature and concentrated in vacuo. Recrystallization of the residue from EtOH/n-hexane gave **2** (9.3 g, 87%) as colorless crystals: mp 75–77 °C (EtOH/*n*-hexane); $[\alpha]_D$ +133 (*c* 1.0, chloroform); ^{1}H NMR ($CDCl_3$) δ 7.48–7.18 (m, 20 H, Ph-H), 5.45 (dd, 1H, $J_{1,2}$ = 9.6 Hz, $J_{2,3}$ = 9.9 Hz, H-2), 4.95 (d, 2H, J = −11.6 Hz, CH_2Ph), 4.60–4.410, (m, 6H, H-1, CH_2Ph), 4.01 (d, 1H, $J_{2,3}$ =

2.8 Hz, H-4), 3.70–3.58 (m, 4H, H-5, 6a, 6b, H-3), 2.98–2.95 (m, 2H, CH_2Ph), 2.79–2.61 (m, 2H, CH_2Ph). ^{13}C NMR ($CDCl_3$) δ 169.9, 168.2 (COO), 86.4 (C-1), 81.5 (C-3), 77.6 (C-5), 74.4, 73.6, 72.1 (CH_2Ph), 72.9 (C-4), 70.4 (C-2), 68.7 (C-6), 28.9, 28.3 ($\mathit{C}H_2COO$); HRMS-FAB (*m*/*z*) calcd for $C_{43}H_{37}O_8SF_5Na^+$: 831.2022; found: 831.2050.

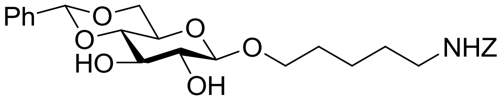

3a

5-(Benzyloxycarbonylamino)pentyl 4,6-*O*-benzylidene-β-D-glucopyranoside (3a)

5-Benzyloxycarbonylaminopentyl β-D-glucopyranoside [2] (3.80 g, 9.52 mmol), benzaldehyde dimethylacetal (1.78 g, 11.61 mmol) and a catalytic amount of *p*-toluenesulfonic acid (0.21 g) were dissolved at 25 °C in MeCN (100 mL), and the resulting solution was stirred until TLC (toluene/acetone 2:1) showed complete consumption of the starting material (ca. 16 h). The mixture was neutralized by the addition of a few drops of triethylamine, diluted with dichloromethane (50 mL), poured into water and extracted with dichloromethane (2 × 70 mL). The combined organic phases were washed with aqueous $NaHCO_3$ solution, dried, filtered and concentrated. The resulting crystalline material was recrystallized from ethyl acetate/*n*-hexane 1:3 to give **3a** (3.30 g, 71%). All physical data were identical to those published previously for compound **3a** [2].

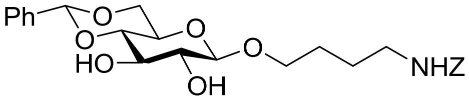

3b

4-(Benzyloxycarbonylamino)butyl 4,6-*O*-benzylidene-β-D-glucopyranoside (3b)

a) TMSOTf (0.14 g, 0.62 mmol) was added under stirring at −10 °C to a solution of 4-(benzyloxycarbonylamino)butanol [3] (1.39 g, 6.22 mmol) and 2,3,4,6-tetra-*O*-benzoyl-α-D-glucopyranosyl trichloroacetimidate [4] (5.07 g, 6.84 mmol) in dichloromethane (100 mL), and the mixture was stirred at −10 °C until TLC indicated that all starting material was consumed (ca. 30 min.). The mixture was neutralized by addition of a few drops of pyridine, washed with aqueous $NaHCO_3$ solution, dried, filtered and concentrated. Chromatography of the residue with toluene/acetone 10:1 afforded 4-(benzyloxycarbonylamino)butyl 2,3,4,6-tetra-*O*-benzoyl-β-D-glucopyranoside (3.8 g, 68%) as a colorless oil. $[\alpha]_D$ +13.2 (*c* 0.5,

$CHCl_3$); ^{1}H NMR ($CDCl_3$) δ 8.02–7.13 (m, 25H, H^{Ph}), 5.89 (t, 1H, $J_{4,5}$ = $J_{3,4}$ = 9.8 Hz, H-4), 5.66 (t, 1H, $J_{2,3}$ = 9.7 Hz, H-3), 5.51 (dd, 1H, $J_{1,2}$ = 7.8 Hz, H-2), 4.79 (d, 1H, H-1), 4.65 (dd, 1H, $J_{6a,6b}$ = −12.0 Hz, H-6a) 4.48 (dd,1H, $J_{5,6b}$ = 12.13 Hz, H-6b), 4.32 (br s, 1H, NH), 4.14–4.10 (m, 1H, H-5), 3.94–3.90 (m, 1H, CH_2), 3.57–3.51 (m, 1H, CH_2), 3.27–3.26 (m, 1H, CH_2), 3.04–3.03 (m, 1H, CH_2), 1.66–1.40 (m, 6H, CH_2); ^{13}C NMR ($CDCl_3$) δ 101.3 (C-1), 78.8 (C-3), 72.2 (C-5), 71.9 (C-2), 69.8 (CH_2Ph), 69.7 (C-4), 66.5 (O CH_2), 63.0 (C-6); ESI-HRMS *m*/*z* calcd for $C_{46}H_{43}O_{12}NNa^+$: 824.26297; found: 824.26256.

b) A few drops of a 1 M solution of NaOMe in MeOH was added at 25 °C to a solution of 4-(benzyloxycarbonylamino)butyl 2,3,4,6-tetra-*O*-benzoyl-β-D-glucopyranoside (3.8 g, 4.67 mmol) in MeOH (100 mL) and the mixture was stirred for 16 h. The mixture was neutralized with Dowex H^+ ion-exchange resin, filtered and concentrated to give crude 4-(benzyloxycarbonylamino)butyl β-D-glucopyranoside (1.8 g, 97%), which was used without further purification in the next step.

c) A solution of 4-(benzyloxycarbonylamino)butyl β-D-glucopyranoside (1.8 g, 4.54 mmol), benzaldehyde dimethylacetal (0.85 g, 5.56 mmol) and a catalytic amount of *p*-toluenesulfonic acid in MeCN (80 mL) was treated as described for the preparation of compound **3a**. Recrystallization of the crystalline residue with ethyl acetate/*n*-hexane 1:1 gave **3b** (1.65 g, 77%). Mp 68–70 °C. $[\alpha]_D$ −38.6 (*c* 0.5, $CHCl_3$). ^{1}H NMR ($CDCl_3$) δ 5.51 (s, 1H, CHPh), 5.09 (s, 2H, CH_2Ph), 4.38 (d, 1H, $J_{1,2}$ = 7.8 Hz, H-1), 4.32 (dd, 1H, H-6a), 3.98–3.95, 3.83–3.73 (2m, 3H, H-5,2,4), 3.57–3.43 (m, 4H, H-3,6b, CH_2O), 3.26–3.24 (m, 2H, CH_2), 1.67–1.62 (m, 4H, CH_2); ^{13}C NMR ($CDCl_3$) δ 156.6 (CO), 103.4 (C-1), 101.8 (CHPh), 80.5 (C-4), 74.6 (C-2), 73.2 (C-3), 70.1 (CH_2O), 68.6 (CH_2O), 66.7 (C-6), 66.4 (C-5), 40,6 (CH_2), 26.9, 26.2 (CH_2); HRMS-FAB (*m*/*z*) calcd for $C_{25}H_{31}O_8NNa^+$: 496.1942; found: 496.2004.

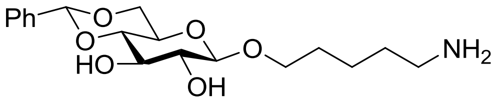

4a

5-Aminopentyl 4,6-*O*-benzylidene-β-D-glucopyranoside (4a)

A suspension of **3a** (1.56 g, 3.2 mmol) and Pd 10% on $BaCO_3$ (ca. 20 mg) in ethanol (60 mL) was vigorously stirred at 25 °C under an atmosphere of hydrogen until TLC (acetone/toluene 3:1) revealed the complete consumption of the starting material (ca. 2 h). The mixture was

filtered through a layer of Celite® and the filtrate is concentrated to give crude **4a** (1.0 g. 91%), which was used for the next step without further purification.

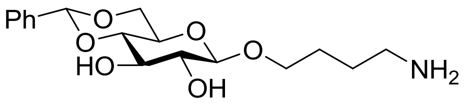

4b

4-Aminobutyl 4,6-*O*-benzylidene-β-D-glucopyranoside (4b)

A suspension of **3b** (0.60 g, 1.56 mmol) and Pd 10% on $BaCO_3$ (ca. 15 mg) in ethanol (30 mL) was treated with hydrogen as described for the preparation of **4a** to give crude **4b** (0.41 g, 80%).

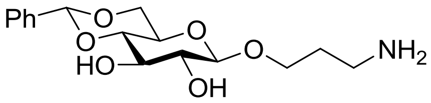

4c

3-Aminopropyl 4,6-*O*-benzylidene-β-D-glucopyranoside (4c)

A suspension of **3c** [5] (0.84 g, 1.83 mmol) and Pd 10% on $BaCO_3$ (ca. 15 mg) in ethanol (40 mL) was treated with hydrogen as described for the preparation of **4a** to give crude **4c** (0.43 g, 73%).

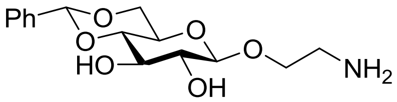

4d

2-Aminoethyl 4,6-*O*-benzylidene-β-D-glucopyranoside (4d)

A suspension of **3d** [6] (0.70 g, 1.56 mmol) and Pd 10% on $BaCO_3$ (ca. 15 mg) in ethanol (30 mL) was treated with hydrogen as described for the preparation of **4a** to give crude **4d** (0.41 g, 87%).

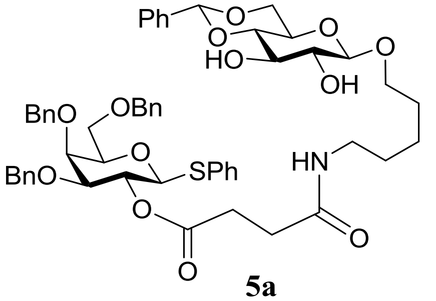

5a

Phenyl 2-*O*-{3-[5-(4,6-*O*-benzylidene-β-D-glucopyranos-1-yloxy)pentylaminocarbonyl]propinonyl}-3,4,6-tri-*O*-benzyl-1-thio-β-*D*-galactopyranoside (5a)

A solution of **2** (1.13 g, 1.4 mmol) and **4a** (1.00 g, 2.91 mmol) in ethyl acetate (100 mL) was stirred at 25 °C until TLC (toluene/acetone 2:1) revealed the total consumption of **2** (16 h). The precipitate, which had formed in the course of the reaction, was filtered off. Recrystallization from acetone/*n*-hexane 1:3 gave **5a** (1.20 g, 88%) as colorless crystals. Mp 158–160 °C. $[\alpha]_D$ −11.2 (*c* 1, $CHCl_3$); ^{1}H NMR ($CDCl_3$) δ 7.43–7.12 (m, 25H, Ph), 6.09 (broad s, 1H, NH), 5.43 (s, 1H, CHPh), 5.27 (broad t, 1H, $J_{1,2}$ = 9.9, $J_{2,3}$ = 9.9, H-2_{gal}), 5.01 (d, 1H, H-1_{gal}), 4.77 (d, 1H, *J* = −11.1, CH_2Ph), 4.59 (d, 1H, *J* = −11.6, CH_2Ph), 4.53 (d, 1H, *J* = −11.6, CH_2Ph), 4.44–4.33 (m, 4H, CH_2Ph, H-1_{glc}), 4.24 (dd, 1H, $J_{5,6a}$ = 4.5, $J_{6a,6b}$ = −10.1, H-$6a_{glc}$), 3.90 (broad s, 1H, H-4_{gal}), 3.77–3.31 (m, 15H, H-$3,5,6a,6b_{gal}$, H-$2,3,4,5,6b_{glc}$, CH_2O, CH_2N, CH_2), 2.73–2.58 (m, 4H, CH_2), 2.45 (t, 2H, CH_2), 1.88–2.00 (m, 2H, CH_2), ^{13}C NMR ($CDCl_3$) δ 172.6 (COO), 171.8 (CONH), 137.9, 137.5, 129.0, 128.4, 128.3, 128.2, 128.1, 127.9, 127.8, 127.4, 126.3 (30 C, Ph), 101.8 (C-1_{glc}), 101.0 (CHPh), 97.6 (C-1_{gal}), 80.4 (C-4_{glc}), 79.7 (C-3_{gal}), 78.8 (C-4_{gal}), 77.6 (C-2_{gal}), 74.3 (C-2_{glc}), 74.1 (CH_2Ph), 73.2 (CH_2Ph), 73.0 (C-3_{glc}), 72.4 (CH_2Ph), 71.7 (C-5_{gal}), 69.0 (CH_2O), 68.7, 68.2 (C-$6_{glc,\ gal}$), 65.5(C-5_{glc}), 38.6 (CH_2N), 31.6, 30.4, 29.7, 28.8, 28.6 (CH_2); HRMS-FAB (*m*/*z*) calcd for $C_{55}H_{63}O_{13}NSNa^+$: 1000.3912; found: 1000.3997.

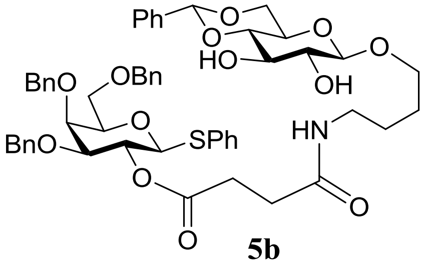

5b

Phenyl 2-*O*-{3-[5-(4,6-*O*-benzylidene-β-D-glucopyranos-1-yloxy)butylaminocarbonyl]propinonyl}-3,4,6-tri-*O*-benzyl-1-thio-β-*D*-galactopyranoside (5b)

A solution of **2** (0.48 g, 0.6 mmol) and **4b** (0.41 g, 1.25 mmol) in ethyl acetate (60 mL) was stirred at 25 °C until TLC (toluene/acetone 2:1) revealed the total consumption of **2** (16 h). The precipitate, which had formed in the course of the reaction, was filtered off. Recrystallization from acetone/*n*-hexane 1:3 gave **5b** (0.45 g, 78%) as colorless crystals. Mp 156–158 °C. $[\alpha]_D$ −8.2 (*c* 1, $CHCl_3$); ^{1}H NMR ($CDCl_3$) δ 7.49–7.16 (m, 25H, Ph), 6.10 (broad t, 1H, J = 5.8, NH), 5.51 (s, 1H, CHPh), 5.43 (t, 1H, $J_{1,2}$ = 9.9, $J_{2,3}$ = 9.9, H-2$_{gal}$), 4.92 (d, 1H, J = −11.5, CH_2Ph), 4.64 (d, 1H, J = −12.0, CH_2Ph), 4.59 (d, 1H, H-1$_{gal}$), 4.56 (d, 1H, J = −11.5, CH_2Ph), 4.52 (d, 1H, J = −12.0, CH_2Ph), 4.44 (d, 1H, J = −11.8, CH_2Ph), 4.39 (d, 1H, J = −11.8, CH_2Ph), 4.36 (d, 1H, $J_{1,2}$ = 7.6, H-1$_{glc}$), 4.31 (dd, 1H, $J_{5,6a}$ = 5.0, $J_{6a,6b}$ = −10.0, H-6a$_{glc}$), 3.97–3.94 (m, 3H, CH_2, H-4$_{gal}$), 3.75 (t, 1H, $J_{2,3}$ = −10.3, $J_{3,4}$ = −10.3, H-3$_{glc}$), 3.64 (dd, 1H, $J_{5,6a}$ = 9.1, $J_{6a,6b}$ = −12.2, H-6a$_{gal}$), 3.60 (broad dd, 2H, H-6b$_{glc,gal}$), 3.56–3.51 (m, 5H, CH_2Ph, OH, H-4$_{glc}$), 3.47 (t, 1H, H-2$_{glc}$), 3.45–3.43 (m, 1H, H-5$_{glc}$), 3.32–3.21 (m, 2H, CH_2O), 2.71–3.52 (m, 2H, CH_2N), 2.49–3.41 (m, 2H, CH_2), 1.66–1.53 (m, 2H, CH_2); ^{13}C NMR δ 172.0 (COO), 171.6 (CONH), 138.3, 137.6, 137.0, 133.4, 131.8, 129.1, 128.7, 128.4, 128.3, 128.0, 127.8, 127.7, 127.5, 127.4, 126.2 (30 C, Ph), 103.3 (C-1$_{glc}$), 101.7 (CHPh), 86.7 (C-1$_{gal}$), 86.6 (C-4$_{glc}$), 81.1 (C-3$_{gal}$), 80.3 (C-4$_{gal}$), 77.5 (C-2$_{gal}$), 74.6 (C-2$_{glc}$), 74.3 (CH_2Ph), 73.5 (CH_2Ph), 73.4 (C-3$_{glc}$), 72.8 (CH_2Ph), 72.0 (C-5$_{gal}$), 70.1 (CH_2O), 69.9, 68.6 (C-6$_{glc, gal}$), 68.5 (C-5$_{glc}$), 39.1 (CH_2N), 31.2, 30.8, 26.4, 25.9 (CH_2); HRMS-FAB (*m*/*z*) calcd for $C_{54}H_{61}O_{13}NSNa^+$: 986.3756; found: 986.3703.

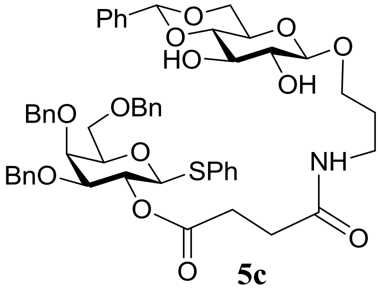

5c

Phenyl 2-*O*-{3-[5-(4,6-*O*-benzylidene-β-D-glucpyranos-1-yloxy)propylaminocabonyl]propinonyl}-3,4,6-tri-*O*-benzyl-1-thio-β-*D*-galactopyranoside (5c)

A solution of **2** (0.89 g, 1.1 mmol) and **4c** (0.72 g, 2.3 mmol) in ethyl acetate (80 mL) was stirred at 25 °C until TLC (toluene/acetone 2:1) revealed the total consumption of **2** (16 h). The precipitate, which had formed in the course of the reaction, was filtered off. Recrystallization from acetone/n-hexane 1:3 gave **5c** (0.94 g, 89%) as colorless crystals. Mp 120–122 °C. $[\alpha]_D$ −10.0 (*c* 0.5, $CHCl_3$); ^{1}H NMR ($CDCl_3$) δ 7.66–7.21 (m, 25H, Ph), 6.35 (broad s, 1H, NH), 5.50 (s, 1H, CHPh), 5.40 (t, 1H, $J_{1,2}$ = 9.6, $J_{2,3}$ = 9.8, H-2$_{gal}$), 4.91 (d, 1H, J = −11.6, CH_2Ph), 4.65–4.51 (m, 3H, CH_2Ph, H-1$_{gal}$), 4.44–4.35 (m, 3H, CH_2Ph, H-1$_{glc}$), 4.30 (dd, 1H, $J_{5,6a}$=5.1, $J_{6a,6b}$ = −10.36, H-6a$_{glc}$), 3.94 (broad s, 1H, H-4$_{gal}$), 3.79–3.22 (m, 13H, H-3,5,6a,6b$_{gal}$, H-2,3,4,5,6b$_{glc}$, CH_2O, CH_2N), 2.72–2.57 (m, 2H, CH_2), 1.76–1.74 (m, 2H, CH_2), 1.26–1.23 (m, 1H, CH_2); ^{13}C NMR ($CDCl_3$) δ 172.1 (COO), 171.8 (CONH), 103.2 (C-1$_{glc}$), 101.8 (CHPh), 86.8 (C-1$_{gal}$, C-4$_{glc}$), 81.1 (C-3$_{gal}$), 80.4 (C-4$_{gal}$), 77.6 (C-2$_{gal}$), 74.7 (C-2$_{glc}$), 74.4 (CH_2Ph), 73.5 (CH_2Ph), 73.4 (C-3$_{glc}$), 72.0 (CH_2Ph), 70.9 (C-5$_{gal}$), 70.2 (CH_2O), 68.7, 68.6 (C-6$_{gal,glc}$), 68.0 (C-5$_{glc}$), 36.7 (CH_2N), 31.3 (CH_2), 30.0 (CH_2), 29.0 (CH_2); HRMS-ESI *m*/*z* calcd for $C_{53}H_{59}O_{13}NSNa^+$: 972.3600; found: 972.3604.

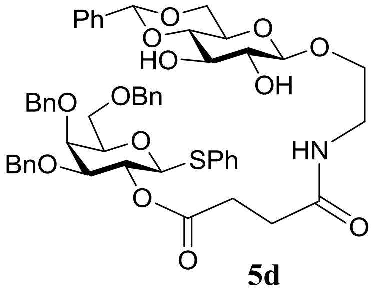

5d

Phenyl 2-*O*-{3-[5-(4,6-*O*-benzylidene-β-D-glucopyranos-1-yloxy)ethylaminocarbonyl]propinonyl}-3,4,6-tri-*O*-benzyl-1-thio-β-*D*-galactopyranoside (5d)

A solution of **2** (0.53 g, 0.66 mmol) and **4d** (0.42 g, 1.36 mmol) in ethyl acetate (40 mL) was stirred at 25 °C until TLC (toluene/acetone 2:1) revealed the total consumption of **2** (16 h). The precipitate, which had formed in the course of the reaction, was filtered off. Recrystallization from acetone/*n*-hexane 1:3 gave **5d** (0.51 g, 83%) as colorless crystals. Mp 165–168 °C. $[\alpha]_D$ −20.0 (*c* 0.5, $CHCl_3$); ^{1}H NMR ($CDCl_3$) δ 7.44–7.20 (m, 25H, Ph), 6.43 (broad t, 1H, NH), 5,38 (s, 1H, CHPh), 5.41 (t, 1H, $J_{1,2}$ = 9.6, $J_{2,3}$ = 9.7, H-2$_{gal}$), 4.85 (d, 1H, J = −11.4, CH_2Ph), 4.65 (d, 1H, J = −11.9, CH_2Ph), 4.59 (d, 1H, J = −11.9, CH_2Ph), 4.58–4.50 (m, 2H, CH_2Ph, H-1$_{gal}$), 4.42 (d, 1H, J = −11.4, CH_2Ph), 4.40–4.36 (m, 2H, CH_2Ph, H-1$_{glc}$), 4.25 (dd, 1H, $J_{5,6a}$ = 5.0, $J_{6a,6b}$ = −10.4, H-6a$_{glc}$), 3.95 (broad s, 1H, H-4$_{gal}$), 3.84–3.19 (m, 11H, H-3,5,6a,6b$_{gal}$, H-2,3,4,5,6b$_{glc}$, CH_2O, CH_2N), 2.91–2.75 (m, 2H, CH_2), 2.67–2.58 (m, 2H, CH_2); ^{13}C NMR ($CDCl_3$) δ 173.8 (COO), 172.5 (CONH), 102.4 (C-1$_{glc}$), 138.3, 138.1, 137.8, 136.9, 129.2, 128.4, 128.3, 128.3, 128.2, 128.1, 128.0, 127.8, 127.7, 127.6, 127.2, 126.2 (30 C, Ph), 101.6 (CHPh), 86.7 (C-1$_{gal}$), 81.2 (C-4$_{glc}$), 80.1 (C-3$_{gal}$), 79.5 (C-4$_{gal}$), 76.2 (C-2$_{gal}$), 74.7 (C-2$_{glc}$), 74.6 (CH_2Ph), 74.4 (CH_2Ph), 73.5 (C-3$_{glc}$), 72.7 (CH_2Ph), 72.5 (C-5$_{gal}$), 69.3 (CH_2O), 68.8, 68.6 (C-6$_{gal,glc}$), 67.8 (C-5$_{glc}$), 38.9 (CH_2N), 25.6, 24.9 (CH_2); HRMS-FAB (*m*/*z*) calcd for $C_{52}H_{57}O_{13}NSNa^+$: 958.3443; found: 958.3517.

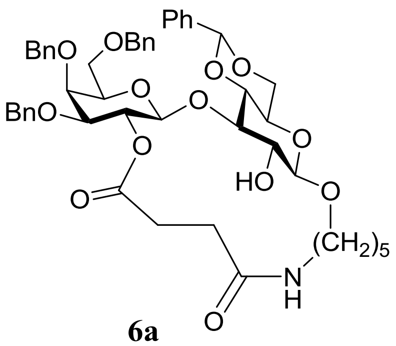

6a

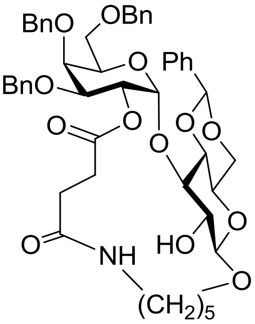

7a

5-Aminopentyl 3,4,5-tri-*O*-benzyl-2-*O*-(3-carboxypropionyl)-β-D-galactopyranosyl-(1→3)-4,6-*O*-benzylidene-β-D-glucopyranoside π-lactam (6a) and 5-aminopentyl 3,4,5-tri-*O*-benzyl-2-*O*-(3-carboxypropionyl)-α-D-galactopyranosyl-(1→3)-4,6-*O*-benzylidene-β-D-glucopyranoside π-lactam (7a)

TMSOTf (121 μL, 0.67 mmol) was added under Ar at −10 °C to a stirred mixture of **5a** (1.2 g, 1.23 mmol) and NIS (1.52 g, 6.7 mmol) in dichloromethane/acetonitrile (1:1, 100 mL), and the mixture was stirred at between −10 and −5 °C until TLC (toluene/acetone 2:1) revealed the complete consumption of **5a** (30–40 min). The mixture was neutralized by the addition of a few drops of triethylamine, brought to 25 °C, diluted with dichloromethane, washed with aqueous $Na_2S_2O_3$ and $NaHCO_3$ solution, dried, filtered and concentrated. Chromatography of the residue with toluene/acetone 4:1 afforded first **6a** (0.54 g, 50%) as colorless crystals. Mp 225–228 °C (acetone/*n*-hexane 1:3). $[\alpha]_D$ −15.0 (*c* 1.0, $CHCl_3$); ^1H NMR ($CDCl_3$) δ 7.47–7.21 (m, 20H, Ph), 5.83 (broad s, 1H, NH), 5.49 (s, 1H, CHPh), 5.31 (dd, 1H, $J_{1,2}$ = 10.0, $J_{2,3}$ = 8.1, H-2_{gal}), 4.89 (d, 1H, J = −11.7, CH_2Ph), 4.63 (d, 1H, H-1_{gal}), 4.62 (d, 1H, J = −11.5, CH_2Ph), 4.62 (d, 1H, CH_2Ph), 4.48 (d, 1H, J = −12.1, $PhCH_2$), 4.38 (m, 1H, H-$6a_{glc}$), 4.34 (d, 1H, $J_{1,2}$ = 7.5, H-1_{glc}), 4.31–4.28 (m, 2H, CH_2Ph), 3.97 (d, 1H, $J_{4,5}$ = 2.5, H-4_{gal}), 3.94–3.90 (m, 1H, OCH_2), 3.74–3.34 (m, 10H, H-3,5,6a,$6b_{gal}$, H-2,3,4,5,$6b_{glc}$, CH_2O), 2.87–2.74 (m, 2H, CH_2), 2.40–2.35 (m, 2H, CH_2), 1.78–1.39 (m, 10H, CH_2); ^{13}C NMR ($CDCl_3$) δ 173.0 (COO), 172.3 (CONH), 138.6, 138.0, 137.8, 137.5, 128.6, 128.4, 128.3, 128.1, 128.0, 127.9, 127.8, 127.7, 127.4, 127.2, 126.2, 126.0 (24 C, Ph), 103.2 (C-1_{gal}), 103.1 (C-1_{glc}), 100.7 (CHPh), 82.9 (C-3_{glc}), 80.0 (C-3_{gal}), 79.6 (C-4_{glc}), 74.5 (CH_2Ph), 73.9 (C-2_{glc}), 73.7 (C-2_{gal}), 73.6 (CH_2Ph), 73.1 (C-5_{gal}), 72.5 (C-4_{gal}), 72.0 (CH_2Ph), 69.4 (CH_2O), 68.7 (C-6_{gal}), 68.0 (C-6_{glc}), 66.0 (C-5_{glc}), 38.9 (CH_2COO), 29.7 (CH_2CON), 29.7, 29.1, 27.1 (CH_2). HRMS-ESI *m/z* calcd for $C_{49}H_{57}O_{13}NNa^+$: 890.3723; found: 890.3727.

Eluted next was **7a** (53 mg, 6%) as colorless crystals. Mp 170–172 °C (acetone/*n*-hexane 1:3). $[\alpha]_D$ −8.6 (*c* 1.0, $CHCl_3$); ^1H NMR ($CDCl_3$) δ 7.49–7.09 (m, 20H, Ph), 6.19 (broad s, 1H, NH) 5.49 (s, 1H, CHPh), 5.32 (dd, 1H, $J_{1,2}$ = 6.0, $J_{2,3}$ = 8.4, H-2_{gal}), 5.08 (d, 1H, H-1_{gal}), 4.85 (d, 1H, *J* = −11.6, CH_2Ph), 4.63 (d, 1H, *J* = −10.4, CH_2Ph), 4.49−4.43 (m, 4 H, CH_2Ph, H-1_{glc}), 4.30 (dd, 1H, $J_{6a,6b}$ = −10.6, H-$6a_{glc}$), 3.95 (broad s, 1H, H-4_{gal}), 3.82−3.37 (m, 11H, H-$3,5,6a,6b_{gal}$, H-$2,3,4,5,6b_{glc}$, CH_2O), 2.92 (d, 1H, CH_2), 2.76−2.69 (m, 1H, CH_2), 2.54−2.39 (m, 2H, CH_2), 2.25–2.20 (m, 1H, CH_2); ^{13}C NMR ($CDCl_3$) δ 171.6 (COO), 170.9 (CONH), 136.8, 136.5, 136.2, 131.5, 128.1, 127.5, 127.4, 127.3, 127.2, 126.9, 126.8, 126.7, 125.3 (24 C, Ph), 101.8 (C-1 $_{glc}$), 100.6 (CHPh), 96.6 (C-1_{gal}), 79.4 (C-3_{gal}), 77.7 (C-3_{glc}), 76.5 (C-4_{glc}), 73.3 (C-2_{gal}), 73.0 (CH_2Ph), 72.5 (CH_2Ph), 72.0 (C-2_{glc}), 71.4 (C-4_{glc}), 71.3 (CH_2Ph), 70.6 (C-5_{gal}), 68.0 (C-6_{glc}), 67.7 (C-6_{gal}), 67.2 (C-5_{glc}), 64.9 (CH_2O), 37.6 (CH_2COO), 30.6 (CH_2N), 29.3, 28.7, 27,6 (CH_2). HRMS-ESI *m/z* calcd for $C_{49}H_{57}O_{13}NNa$: 890.3723; found: 890.3721.

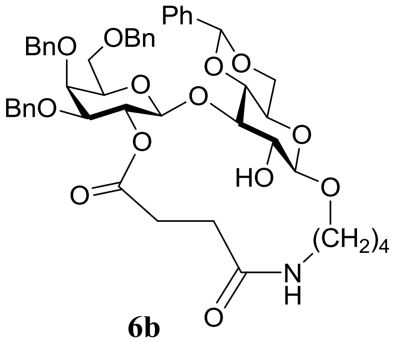

6b

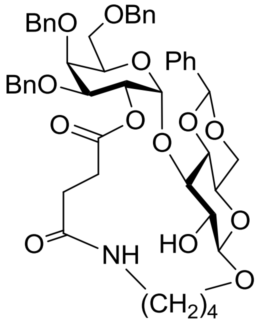

7b

4-Aminobutyl 3,4,5-tri-*O*-benzyl-2-*O*-(3-carboxypropionyl)-β-D-galactopyranosyl-(1→3)-4,6-*O*-benzylidene-β-D-glucopyranoside ο-lactam (6b) and 4-aminobutyl 3,4,5-tri-*O*-benzyl-2-*O*-(3-carboxypropionyl)-α-D-galactopyranosyl-(1→3)-4,6-*O*-benzylidene-β-D-glucopyranoside ο-lactam (7b)

Treatment of a suspension of **5b** (0.41 g, 0.43 mmol) and NIS (0.53 g, 2.34 mmol) in dichloromethane (30 mL) with TMSOTf (42 μL, 0.23 mmol) at −10 °C as described for the preparation of **6a** followed by chromatography with dichloromethane/acetone/toluene 4:1:1 gave first **6b** (0.21 g, 58%) as colorless crystals. Mp 210–212 °C (acetone/*n*-hexane 1:3). $[\alpha]_D$ −13.0 (*c* 1.0, $CHCl_3$); ^1H NMR ($CDCl_3$) δ 7.73–7.23 (m, 20H, Ph), 6.04 (broad s, 1H, NH), 5.51 (s, 1H, CHPh), 5.39 (dd, 1H, $J_{1,2}$ = 7.8, $J_{2,3}$ = 9.9, H-2_{gal}), 4.92 (d, 1H, *J* = −11.6, CH_2Ph), 4.74 (d, 1H, H-1_{gal}), 4.65–4.56 (m, 7H, CH_2Ph, H-$1,6a_{glc}$), 3.97 (d, 1H, $J_{4,5}$ = 3.3, H-4_{gal}), 3.91–3.38 (m, 11H, H-$2,3,4,5,6b_{glc}$, H-$3,5,6a,6b_{gal}$, CH_2O), 3.07–3.04 (m, 2H, CH_2N), 2.79–2.65 (m, 8H, CH_2); ^{13}C NMR ($CDCl_3$) δ 173.0 (COO), 172.3 (CONH), 138.5–125.3 (24

C, Ph), 102.8 (C-1_{gal}), 102.6 (C-1_{glc}), 100.6 (CHPh), 82.6 (C-3_{glc}), 80.1 (C-3_{gal}), 79.8 (C-4_{glc}), 74.4 (CH_2Ph), 73.8 (C-4 $_{gal}$), 73.5 (2 C, CH_2Ph, C-5_{gal}), 72.7 (C-2_{gal}), 72.1 (C-2_{glc}), 71.8 (CH_2Ph) 68.6 (C-6_{gal}), 68.1 (C-6_{glc}), 67.7 (CH_2O), 66.6 (C-5_{glc}), 26.3, 29.6, 30.4, 38.9 (CH_2); HRMS-FAB (*m*/*z*) calcd for $C_{48}H_{55}O_{13}NNa^+$: 876.3566; found: 876.3580.

Eluted next was **7b** (40 mg, 8%) as colorless crystals. Mp 205–207 °C (acetone/*n*-hexane 1:3). $[\alpha]_D$ −23.0 (*c* 1.0, $CHCl_3$); ^{1}H NMR ($CDCl_3$) δ 7.48–7.24 (m, 20H, Ph), 5.85 (broad s, 1H, NH), 5.72 (d, 1H, $J_{1,2}$ = 3.9, H-1_{gal}), 5.41 (s, 1H, CHPh), 5,22 (d, 1H, $J_{2,3}$ = 10.6, H-2_{gal}), 4.90 (d, 1H, *J* = −11.5, CH_2Ph), 4.72–4.40 (m, 8H, CH_2Ph, H-1_{glc}), 4.29 (dd, 1H, $J_{6a,6b}$ = −10.2, H-$6a_{glc}$), 4.00 (d, 1H, $J_{4,5}$= 4.9, H-4_{gal}), 3.83–3.49 (m, 11H, H-$3,5,6a,6b_{gal}$, H-$2,3,4,5,6b_{glc}$, CH_2O), 3.06–2.97 (m, 1H, CH_2), 2.83–2.79 (broad d, 1H, CH_2), 2.57–2.25 (m, 4H, CH_2), 1.68–1.51 (m, 8H, CH_2); ^{13}C NMR ($CDCl_3$) δ 172.9 (COO), 172.1 (CONH), 138.4–124.6 (24 C, Ph), 104.0 (C-1_{glc}), 101.6 (CHPh), 94.7 (C-1_{gal}), 81.2 (C-3_{gal}), 76.9 (C-4_{gal}), 75.7 (C-4_{glc}), 74.6 (CH_2Ph), 73.5 (C-3_{glc}), 72.8 (CH_2Ph), 72.6 (C-2_{glc}), 72.3 (CH_2Ph), 72.0 (C-2_{gal}), 69.8 (C-6_{glc}), 69.3 (C-6_{gal}), 68.7 (CH_2O), 68.6 (C-5_{gal}), 65.5 (C-5_{glc}); HRMS-FAB (*m*/*z*) calcd for $C_{48}H_{55}O_{13}NNa^+$: 876.3566; found: 876.3597.

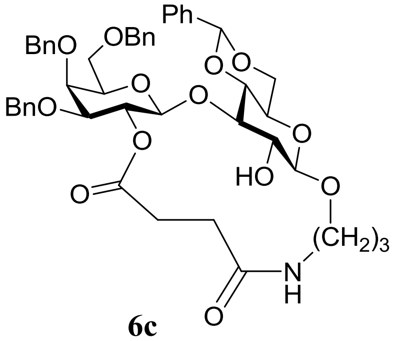

6c

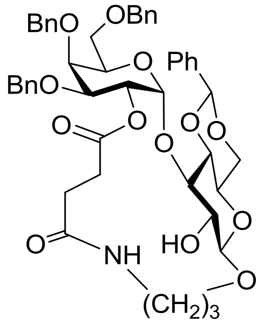

7c

3-Aminopropyl 3,4,5-tri-*O*-benzyl-2-*O*-(3-carboxypropionyl)-β-D-galactopyranosyl-(1→3)-4,6-*O*-benzylidene-β-D-glucopyranoside ξ-lactam (6c) and 3-aminopropyl 3,4,5-tri-*O*-benzyl-2-*O*-(3-carboxypropionyl)-α-D-galactopyranosyl-(1→3)-4,6-*O*-benzylidene-β-D-glucopyranoside ξ-lactam (7c)

Treatment of a suspension of **5c** (0.65 g, 0.68 mmol) and NIS (0.53 g, 2.34 mmol) in dichloromethane/acetonitrile (60 mL) with TMSOTf (42 μL, 0.23 mmol) at −5 °C as described for the preparation of **6a** followed by chromatography with dichloromethane/acetone/toluene 5:2:1 gave first **6c** (0.33 g, 58%) as colorless crystals. Mp

218–221 °C (acetone/*n*-hexane 1:3). $[\alpha]_D$ −11.0 (*c* 1.0, $CHCl_3$); ^1H NMR ($CDCl_3$) δ 7.48–7.18 (m, 20H, Ph), 5.95 (broad d, 1H, NH), 5.50 (s, 1H, CHPh), 5.25 (dd, 1H, $J_{2,3}$ = 7.6, $J_{3,4}$ = 7.8, H-2_{gal}), 4.74–4.53 (m, 6H, H-1_{gal}, H-1_{glc}, CH_2Ph), 4.41 (t, 2H, J = −11.9, CH_2Ph), 4.31 (dd, 1H, $J_{6a,6b}$ = −10.4, H-$6a_{glc}$), 3.98 (d, 1H, J = 2.5, H-4_{gal}), 3.74–3.33 (m, 11H, H-3,5,6a,$6b_{gal}$, H-2,3,4,5,$6b_{glc}$, CH_2O), 3.04–2.87 (m, 1H, CH_2), 2.88–2.80 (m, 2H,CH_2), 2.43–2.34 (m, 2H,CH_2), 2.45–2.28 (m, 2H,CH_2); ^{13}C NMR ($CDCl_3$) δ 173.2 (COO), 172.6 (CONH), 137.8–126.3 (24 C, Ph), 102.7 (C-1_{gal}), 102.2 (C-1_{glc}), 100.3 (CHPh), 82.3 (C-3_{glc}), 79.9 (C-3_{gal}), 78.7 (C-4_{glc}), 74.3 (CH_2Ph), 74.1 (C-2_{gal}), 73.6 (CH_2Ph), 73.1 (C-5_{gal}), 72.8 (C-4_{gal}), 72.3 (CH_2Ph), 70.8 (C-2_{glc}), 68.5 (C-6_{glc}), 68.2 (C-6_{gal}), 66.3 (C-5_{glc}); HRMS-FAB (*m/z*) calcd for $C_{47}H_{53}O_{13}NNa^+$: 862.3410; found: 862.3445.

Eluted next was **7c** contaminated by traces of **6c** (90 mg, 15%) as amorphous solid. ^1H NMR ($CDCl_3$) δ 7.48–7.18 (m, 20H, Ph), 5.95 (broad d, 1H, NH), 5.50 (s, 1H, CHPh), 5.25 (dd, 1H, $J_{2,3}$ = 7.6, $J_{3,4}$ = 7.9, H-2_{gal}), 4.74–4.53 (m, 6H, H-1_{gal}, H-1_{glc}, CH_2Ph), 4.45 (t, 2H, J = −11.9, CH_2Ph), 4.31 (dd, 1H, $J_{6a,6b}$ = −10.4, H-$6a_{glc}$), 3.98 (d, 1H, $J_{4,5}$ = 2.5, H-4_{gal}), 3.74–3.33 (m, 11H, H-3,6a,$6b_{gal}$, H-2,3,4,5,$6b_{glc}$, CH_2O), 3.04–2.87 (m, 1H, CH_2), 2.88–2.80 (m, 2H, CH_2), 2.43–2.34 (m, 2H, CH_2), 2.45–2.28 (m, 2H, CH_2); ^{13}C NMR ($CDCl_3$) δ 173.2 (COO), 172.6 (CONH), 102.2 (C-1_{glc}), 100.3 (CHPh), 95.4 (C-1_{gal}), 82.3 (C-3_{glc}), 79.9 (C-3_{gal}), 78.7 (C-4_{glc}), 74.3 (CH_2Ph), 74.1 (C-2_{gal}), 73.6 (CH_2Ph), 73.1 (C-5_{gal}), 72.8 (C-4_{gal}), 72.3 (CH_2Ph), 70.8 (C-2_{glc}), 68.5 (C-6_{glc}), 68.2 (C-6_{gal}), 66.3 (C-5_{glc}).

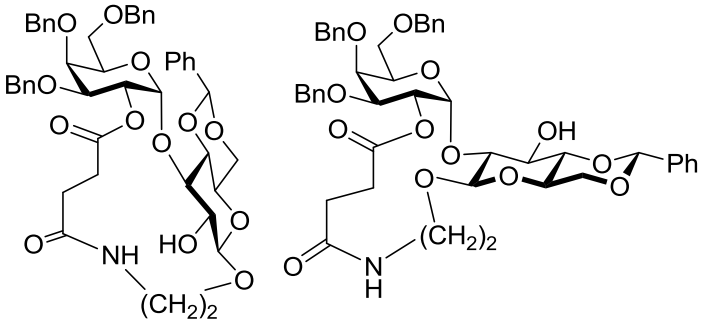

7d **8d**

2-Aminoethyl 3,4,5-tri-*O*-benzyl-2-*O*-(3-carboxypropionyl)-α-D-galactopyranosyl-(1→3)-4,6-*O*-benzylidene-β-D-glucopyranoside ν-lactam (7d) and 2-aminoethyl 3,4,5-tri-*O*-benzyl-2-*O*-(3-carboxypropionyl)-α-D-galactopyranosyl-(1→2)-4,6-*O*-benzylidene-β-D-glucopyranoside μ-lactam (8d)

Treatment of a suspension of **5d** (0.44 g, 0.47 mmol) and NIS (0.58 g, 2.57 mmol) in dichloromethane/acetonitrile (60 mL) with TMSOTf (46 μL, 0.26 mmol) at −5 °C as described for the preparation of **6a** followed by chromatography with dichloromethane/acetone/toluene 5:2:1 gave first **8d** (0.23 g, 59%) as colorless crystals. Mp 209–212 °C (acetone/*n*-hexane 1:3). $[\alpha]_D$ +6.0 (*c* 1.0, $CHCl_3$); ^1H NMR ($CDCl_3$) δ 7.50–7.24 (m, 20H, Ph), 6.35 (broad d, 1H, NH), 5.54 (s, 1H, CHPh), 5.50 (dd, 1H, $J_{2,3}$ = 9.7, $J_{3,4}$ = 9.7, H-2_{gal}), 4.84 (br dd, 2H, CH_2Ph, H-1_{gal}), 4.61 (br dd, 2H, CH_2Ph), 4.46–4.40 (m, 3H, CH_2Ph, H-1_{glc}), 4.35–4.29 (m, 3H, CH_2Ph, H-$6a_{glc}$), 3.96 (t, 1H, $J_{2,3}$ = 9.3, $J_{3,4}$ = 9.3, H-3_{gal}), 3.91 (d, 1H, $J_{4,5}$ = 2.7, H-4_{gal}), 3.84–3.36 (m, 12H, H-5,6a,$6b_{gal}$, H-2,3,4,5,$6b_{glc}$, CH_2O), 3.01–2.95 (m, 1H, CH_2), 2.77–2.67 (m, 1H, CH_2), 2.58–2.42 (m, 2H, CH_2), 2.27–2.22 (m, 1H, CH_2); ^{13}C NMR ($CDCl_3$) δ 172.7 (COO), 172.1 (CONH), 137.9–126.5 (24 C, Ph), 101.9 (CHPh), 99.9 (C-1_{glc}), 97.3 (C-1_{gal}), 81.0 (C-3_{glc}), 80.5 (C-2_{glc}), 80.1 (C-4_{glc}), 74.4 (CH_2Ph), 74.1 (C-5_{gal}), 73.7 (CH_2Ph), 72.9 (CH_2Ph), 71.5 (C-4_{gal}), 70.9 (C-2_{gal}), 70.9 (C-3_{gal}), 68.6 (C-6_{glc}), 68.1 (C-6_{gal}), 67.0 (CH_2O), 65.8 (C-5_{glc}), 39.2 (CH_2), 31.9 (CH_2), 31.7 (CH_2), 31.0 (CH_2); HRMS-FAB (*m*/*z*) calcd for $C_{46}H_{51}O_{13}NNa^+$: 848.3253; found: 848.3288.

Eluted next was **7d** (50 mg, 13%) as colorless crystals. Mp 148–150 °C (acetone/*n*-hexane 1:3). $[\alpha]_D$ +2.0 (*c* 1.0, $CHCl_3$); ^1H NMR ($CDCl_3$) δ 7.50–7.25 (m, 20H, Ph), 6.35 (br d, 1H, NH), 5.54 (s, 1H, CHPh), 5.50 (t, 1H, $J_{2,3}$ = 9.1, $J_{3,4}$ = 9.1, H-2_{gal}), 4.87 (d, 1H, J = −11.9, CH_2Ph), 4.82 (d, 1H, $J_{1,2}$= 7.8, H-1_{gal}), 4.64–4.56 (m, 2H, CH_2Ph), 4.47–4.41 (m, 2H, CH_2Ph, H-1_{glc}), 4.32 (dd, 1H, $J_{6a,6b}$ = −13.1, H-$6a_{glc}$), 3.97 (t, 1H, $J_{3,4}$=8.8, H-3_{gal}), 3.92 (d, 1H, $J_{4,5}$ =

1.5, H-4$_{gal}$), 3.85–3.36 (m, 10H, H-5,6a,6b$_{gal}$, H-2,3,4,5,6b$_{glc}$, CH_2O), 3.02–2.95 (m, 1H, CH_2O), 2.77–2.68 (m, 1H, CH_2), 2.59–2.43 (m, 2H, CH_2), 2.28–2-23 (m, 1H, CH_2); ^{13}C NMR ($CDCl_3$) δ 172.6 (COO), 172.0 (CONH), 137.9–126.5 (24 C, Ph), 101.8 (CHPh), 99.8 (C-1$_{glc}$), 97.2 (C-1$_{gal}$), 80.9 (C-3$_{glc}$), 80.4 (C-2$_{glc}$), 80.0 (C-4$_{glc}$), 74.7 (C-5$_{gal}$), 74.3 (CH_2Ph), 74.0 (CH_2Ph), 72.1 (CH_2Ph), 71.3 (C-4$_{gal}$), 70.9 (C-3$_{gal}$), 70.8 (C-2$_{gal}$), 68.5 (C-6$_{gal}$), 68.0 (CH_2O), 66.9 (C-6$_{glc}$), 65.7 (C-5$_{glc}$), 39.1 (CH_2), 31.8, 31.6, 30.9 (CH_2); HRMS-FAB (*m*/*z*) calcd for $C_{46}H_{51}O_{13}NNa^+$: 848.3253; found: 848.3205.

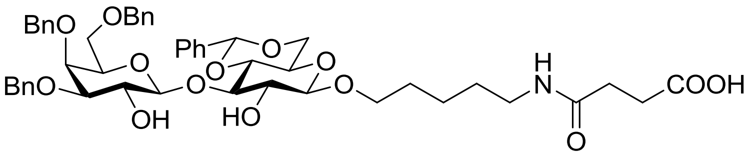

9

5-(3-Hydroxycarbonylpropanamido)pentyl 3,4,6-tri-*O*-benzyl-β-D-galactopyranosyl-(1→3)-4,6-*O*-benzylidene-β-D-glucopyranoside (9)

A few drops of a 1 M solution of NaOMe in MeOH was added at 50 °C to a solution of **6a** (0.35 g, 0.40 mmol) in toluene/MeOH 1:1 (20 mL), and the solution was stirred at 60 °C for 3 h. The solution was cooled to rt, neutralized with Dowex 50W X8 ion-exchange resin (H^+ form), filtered and concentrated. Chromatography of the residue with toluene/MeOH 3:1 gave **9** (0.30 g, 84%) as a colorless amorphous solid. $[\alpha]_D$ −16.0 (*c* 1.0, $CHCl_3$); ^{1}H NMR ($CDCl_3$) δ 7.49–7.23 (m, 20H, Ph), 5.94 (d, 1H, *J* = 7.8, H-1$_{gal}$), 5.63 (s, 1H, CHPh), 4.87 (d, 1H, *J* = −11.1, CH_2Ph), 4.57–4.44 (m, 3H, CH_2Ph, H-1$_{glc}$), 4.27 (brs, 2H, NH), 4.08 (br s, 1H, H-4$_{gal}$), 3.79–3.08 (m, 17H, H-2,3,5,6a,6b, OH$_{gal}$, H-2,3,4,5,6a,6b,OH$_{glc}$, CH_2O, CH_2), 2.41–2.35 (m, 2H,CH_2), 1.70–1.57 (m, 6H, CH_2); ^{13}C NMR ($CDCl_3$) δ 172.4 (CONH), 157.7 (CO), 105.8 (C-1$_{gal}$), 103.6 (C-1$_{glc}$), 100.4 (CHPh), 83.6 (C-3$_{glc}$), 82.5 (C-3$_{gal}$), 79.4 (C-4$_{glc}$) 75.0 (CH_2Ph), 74.6 (C-2$_{gal}$), 73.4 (C-2$_{glc}$), 73.3 (CH_2Ph), 72.5 (C-4$_{gal}$), 72.4 (CH_2Ph), 71.2 (C-5$_{gal}$), 69.8 (C-6$_{gal}$), 69.8 (C-6$_{glc}$), 67.9 (CH_2O), 66.6 (C-5$_{glc}$), 34.3, 31.9, 31.6, 29.8, 26.3, 25.4, 23.7 (CH_2); HRMS-ESI *m*/*z* calcd for $C_{49}H_{59}O_{14}NNa^+$: 908.38283; found: 908.38278.

References

1. Ziegler, T.; Dettmann, R.; Ariffadhillah; Zettl, U. *J. Carbohydr. Chem.* **1999,** *18,* 1079–1095. doi:10.1080/07328309908544056
2. Eckhardt, E.; Ziegler, T. *Carbohydr. Res.* **1994,** *264,* 253–269. doi:10.1016/S0008-6215(05)80010-2
3. Sengupta, D.; Blaskó, A.; Bruice, T. C. *Bioorg. Med. Chem.* **1996,** *4,* 803–813. doi:10.1016/0968-0896(96)00070-3
4. Ziegler, T.; Jurisch, K. *Tetrahedron: Asymmetry* **2000,** *11,* 3403–3418. doi:10.1016/S0957-4166(00)00294-9
5. Wang, Y.; Yan, Q.; Wu, J.; Zhang, L.-H.; Ye, X.-S. *Tetrahedron* **2005,** *61,* 4313–4321. doi:10.1016/j.tet.2005.02.023
6. Hanessian, S.; Tremblay, M.; Kornienko, A.; Moitessier, N. *Tetrahedron* **2001,** *57,* 3255–3265. doi:10.1016/S0040-4020(01)00194-6
